# The association between the lipid profile and fasting blood glucose with weight related outcomes in healthy obese adults

**DOI:** 10.1186/s13104-018-3485-4

**Published:** 2018-06-14

**Authors:** Shirley Telles, Sushma Pal, Sachin Kumar Sharma, Alok Singh, Niranjan Kala, Acharya Balkrishna

**Affiliations:** Patanjali Research Foundation, Patanjali Yogpeeth, Haridwar, Uttarakhand 249405 India

**Keywords:** Obese adults, Fasting blood glucose, BMI, Waist circumference, Lipid profile, Regression analysis

## Abstract

**Objectives:**

The present study was conducted on healthy obese persons to determine: (i) the association between total cholesterol, triglycerides, HDL cholesterol, total cholesterol/HDL ratio and fasting blood glucose (FBG) with (a) BMI, (b) waist circumference (WC) and (c) body fat and (ii) the presence of undiagnosed type 2 diabetes (based on fasting blood glucose) in the participants. There were 1140 participants of both sexes (female:male 697:443; group mean age 44.0 ± 10.8 years; BMI ≥ 25 kg/m^2^) from four regions of India. The participants were assessed for (i) BMI and WC, (ii) body fat, (iii) fasting serum lipid profile and (iv) FBG. Statistical significance (α) was set at 0.05.

**Results:**

Based on a linear regression analysis triglycerides acted as a significant predictor for body fat. Triglycerides showed a significant negative correlation with BMI and body fat. HDL cholesterol was significantly negatively correlated with waist circumference and positively correlated with body fat. Total cholesterol/HDL ratio was positively correlated with waist circumference and negatively correlated with body fat. A significant positive correlation of FBG and waist circumference was also observed. Among the healthy participants 34.2% had pre-diabetes and 13.6% had diabetes.

## Introduction

Obesity has increased worldwide with an increase in the incidence of associated diseases especially type 2 diabetes mellitus (DM). Central adiposity is considered particularly dangerous as visceral fat is known to secrete pro-inflammatory substances and is associated with an increase in dyslipidemia and cardiovascular disease [1].

Evidence from epidemiological studies suggests that the relationship between obesity and developing cardiovascular disease begins early in life [2]. Autopsy reports on adolescents who died in accidents found an association between fatty streaks in the coronary arteries and aorta with abnormal trends in the lipid profile, blood pressure and body weight prior to death [2]. Apart from this epidemiological evidence a longitudinal study on young adults (aged between 19 and 32 years) who were overweight, hypertensive and had dyslipidemia had the same risk factors (i.e., being overweight, with elevated blood pressure levels and abnormal serum lipid profile values) in childhood [2]. These results suggest the importance of understanding the association between the body weight and lipid profile in persons who are obese to consider ways for management.

Multiple factors contribute to the risk of persons who are obese developing cardiovascular and other diseases. These factors include the BMI, central obesity and also raised fasting blood glucose levels. A study on 10,913 healthy persons, who were arbitrarily categorized based on their FPG levels as 5 groups (i.e., 50–79, 80–84, 85–89, 90–94, and 95–99 mg/dL) were followed up for an average of 4.3 years showed that participants who had fasting glucose levels in the high normal range (95–99 mg/dL) had an increased risk of cardiovascular disease when compared with other four groups [3].

Two hundred and four obese persons of both sexes from west India were assessed to determine the incidence of type 2 diabetes mellitus among them and to determine if the fasting blood glucose (FBG) levels had any correlation with the body mass index (BMI) and waist circumference (WC). Thirty percent of overweight persons (BMI > 23 kg/m^2^) and 25% of obese persons (BMI > 25 kg/m^2^) were diabetic and a positive correlation was found between the BMI and WC [4]. Random blood glucose was positively correlated with WC in a Nigerian population [5], while another study correlated FBG positively with WC in Iran [6].

With this background on the importance of dyslipidemia and raised fasting blood glucose levels as risk factors for cardiovascular disease in the obese, the present study was conducted on 1140 obese persons to determine if this sample drawn from four regions in India (north, south, east and west) would show whether (i) total cholesterol, triglycerides, HDL cholesterol and total cholesterol/HDL ratio could predict the BMI, WC and body fat, (ii) any association between the fasting blood glucose with the BMI, WC and body fat and (iii) the presence of diabetes (based on the fasting blood glucose levels) unknown to them, as presence of diagnosed diabetes was one of the exclusion criteria.

## Main text

### Methods

#### Study design and recruitment of research participants

One thousand one hundred and forty obese persons of both sexes were recruited for the study from four regions of India (female:male 697:443; group mean age ± SD 44.0 ± 10.8 years). They were from north (60.2%), south (12.5%), east (12.1%) and west (15.2%) regions of India based on a standard categorization of the regions [7]. Participants were included in the trial if they met the following criteria (i) BMI ≥ 25 kg/m^2^ and (ii) not currently taking part in a weight loss program. Participants were excluded if (i) they had any diseases such as type 2 DM, cardiovascular disease or any other condition associated with obesity and (ii) if obesity was secondary to any medical condition or medication (e.g., steroids). Recruitment was by social workers connected to the research institution conducting the trial, who were located in the four zones. All participants gave their signed consent to participate. The study had the ethical approval of the Ethics Committee of Patanjali Research Foundation, Haridwar, India (Approval No. YRD-017/022).

The assessments included (i) standard anthropometry (i.e., BMI, WC), (ii) body composition using an electrical impedance analyzer (BF-907, Maltron, UK) to assess body fat and (iii) serum lipid profile (from the fasting sample) using appropriate enzymes followed by spectrophotometry and (iv) fasting blood glucose based on a blood sample drawn from the right antecubital vein after an overnight fast, most often with the night meal at 20:00 h and the sample drawn after 08:00 h the next day. FBG was estimated by spectrophotometry. A methodological limitation is that the conventional two consecutive FBG samples were not obtained.

#### Data analysis

Data were analyzed using SPSS (Version 24.0).

Multiple linear regression analyses was performed to determine if lipid levels (total cholesterol, triglycerides, HDL cholesterol and total cholesterol/HDL ratio) could predict weight related variables (BMI, waist circumference and body fat). Three separate linear regression analyses were carried out with total cholesterol, triglycerides, HDL cholesterol and total cholesterol/HDL ratio as independent variables. In each case one of the following, that is (i) BMI, (ii) waist circumference and (iii) body fat acted as a dependent variable.

To determine the relationship between (i) total cholesterol, triglycerides, HDL cholesterol and total cholesterol/HDL ratio with weight related variables (BMI, body fat and waist circumference) and (ii) FBG with the weight related variables mentioned above, the Pearson correlation test was used.

### Results

Group mean values ± SD for all variables are given in Table 1.

**Table 1:** The group mean values ± SD of different variables

Variables	Mean ± SD
BMI (kg/m^2^)	31.8 ± 4.9
Waist circumference (cm)	104.6 ± 13.7
Body fat (kg)	34.1 ± 11.5
Cholesterol (mmol/L)	4.57 ± 0.92
Triglycerides (mmol/L)	1.68 ± 0.91
High density lipoprotein (mmol/L)	1.15 ± 0.29
Cho/HDL ratio	4.2 ± 1.2
Fasting blood glucose (mmol/L)	5.88 ± 1.87

*BMI* body mass index, *Cho* cholesterol, *HDL* high density lipoprotein, *SD* standard deviation

#### Regression results

Based on linear regression triglycerides acted as a significant predictor for body fat {adjusted R^2^ = 0.006; beta coefficient = − 0.095; P = 0.019; 95% CI of [− 0.002, − 0.022]; tolerance =0.532; variance inflation factors (VIF) = 1.880}.

#### Correlation results

Triglycerides showed a significant negative correlation with (i) BMI (P = 0.007, r = − 0.073) and (ii) body fat (P = 0.001, r = − 0.089). HDL cholesterol was significantly negatively correlated with waist circumference (P < 0.001, r = − 0.110) and positively correlated with body fat (P = 0.008, r = 0.072). Total cholesterol/HDL ratio were positively correlated with waist circumference (P = 0.004, r = 0.078) and negatively correlated with body fat (P = 0.038, r = − 0.053). A significant positive correlation of FBG with waist circumference was observed (P < 0.001, r = 0.114).

### Discussion

Most previous studies have shown that levels of triglycerides are positively associated with the BMI and body fat in obese persons [8, 9]. However in the present population of 1140 healthy obese adults the findings were contrary to the earlier studies [8, 9]. In the present study the levels of triglycerides were negatively correlated with the BMI and body fat and acted as a negative predictor for body fat. This difference in the findings may be related to two factors (i) 70% of the participants were plant-based lacto vegetarians (based on their self-report), which is associated with lower cholesterol and triglycerides levels [10, 11] and (ii) a difference in the level of physical activity between participants of the earlier studies and those of the present study. Earlier studies were carried out on sedentary obese persons who had high triglycerides and low HDL cholesterol levels. While in the present study 63.0% of the participants had normal triglycerides (> 1.7 mmol/L) and HDL levels (on an average 1.2 mmol/L for males and 1.3 mmol/L for females) though their BMI (on an average 33 kg/m^2^) and body fat (on an average 40%) were high. High triglycerides levels and low HDL levels have been attributed to a sedentary lifestyle [12, 13]. Based on the normal triglycerides and HDL levels in the present obese population it could be speculated that the population of the present study could be more physically active compared to those of the earlier studies. This may explain the results of the present study. However in the absence of details of physical activity it remains only a speculation.

The negative relationship of waist circumference with HDL levels and positive relationship with total cholesterol/HDL ratio resembles the findings of the earlier studies which were carried out to determine the relation between waist circumference (WC) and lipid levels [14].

The fasting blood glucose (FBG) showed a positive correlation with the WC. This is in line with a clinical report which states that raised FBG levels within the normal range are associated with a higher risk for cardiovascular disease than the association with serum lipid levels [3]. Also among the total sample (n = 1140), 13.6% had higher fasting blood glucose levels i.e., higher than > 7.0 mmol/L, though all participants were supposedly healthy. Hence unknown to them 13.6% of the participants had higher than normal FBG levels which could lead to a diagnosis of type 2 DM on further investigation.

In obese adult south Asians given the complex interaction between a genetic predisposition to central obesity, type 2 DM as well as a greater likelihood of consuming a lacto-vegetarian diet with high carbohydrate content [3] it is essential to have details about the diet and physical activity, anthropometry, body composition, the lipid profile and FBG.

## Limitations

The study was a single group trial. This is a major limitation of the study. Also, the findings (a negative correlation between triglycerides with the BMI and body fat) have been attributed to the participants’ diet and level of physical activity. However a more detailed record of diet and level of physical activity would be useful.

## Abbreviations

BMI: body mass index
CI: confidence interval
DM: diabetes mellitus
FBG: fasting blood glucose
HDL: high density lipoprotein
SD: standard deviation
SPSS: Statistical Package for Social Sciences
WC: waist circumference

## References

[1] Després JP, Lemieux I (2006). Abdominal obesity and metabolic syndrome. Nature.

[2] Plourde G (2002). Impact of obesity on glucose and lipid profiles in adolescents at different age groups in relation to adulthood. BMC Fam Pract.

[3] Shaye K, Amir T, Shlomo S, Yechezkel S (2012). Fasting glucose levels within the high normal range predict cardiovascular outcome. Am Heart J.

[4] Patil SP, Sukumaran S, Bhate A, Mukherji A, Chandrakar S (2012). Correlation of blood sugar with waist circumference and body mass index in an Indian population. Glob J Pharm.

[5] Etukumana EA, Puepet FH, Obadofin MO (2014). Relationship of blood glucose levels with waist circumference, hip circumference and waist-hip ratio among rural adults in Nigeria. Asian J Pharm Clin Res.

[6] Veghari G, Sedaghat M, Joshaghani H, Banihashem S, Moharloei P, Angizeh A (2014). The association of fasting blood glucose and waist circumference in northern adults in Iran: a population based study. J Diabetes Metab Disord.

[7] List of regions-zone-wise, division and state-wise. https://www.licindia.in/…/Empanelment-of-TPAs-for-providing-services-for-LIC. Accessed 14 Mar 2018.

[8] Hu D, Hannah J, Gray RS, Jablonski KA, Henderson JA, Robbins DC (2000). Effects of obesity and body fat distribution on lipids and lipoproteins in nondiabetic American Indians: the strong heart study. Obes Res.

[9] Shamai L, Lurix E, Shen M, Novaro GM, Szomstein S, Rosenthal R (2011). Association of body mass index and lipid profiles: evaluation of a broad spectrum of body mass index patients including the morbidly obese. Obes Surg.

[10] National Institute of Nutrition (2011). Dietary guidelines for Indians-A manual.

[11] Norris J. Cardiovascular disease markers in Vegans. https://veganhealth.org/cardiovascular-disease-markers-in-vegans/. Accessed 5 May 2018.

[12] Skoumas J, Pitsavos C, Panagiotakos DB, Chrysohoou C, Zeimbekis A, Papaioannou I (2003). Physical activity, high density lipoprotein cholesterol and other lipids levels, in men and women from the ATTICA study. Lipids Health Dis.

[13] Crichton GE, Alkerwi A (2015). Physical activity, sedentary behavior time and lipid levels in the observation of cardiovascular risk factors in Luxembourg study. Lipids Health Dis.

[14] Chehrei A, Sadrnia S, Keshteli AH, Daneshmand MA, Rezaei J (2007). Correlation of dyslipidemia with waist to height ratio, waist circumference, and body mass index in Iranian adults. Asia Pac J Clin Nutr.

